# Editorial: Fibrosis and Inflammation in Tissue Pathophysiology

**DOI:** 10.3389/fphys.2021.830683

**Published:** 2022-01-21

**Authors:** Isotta Chimenti, Susanne Sattler, Gonzalo del Monte-Nieto, Elvira Forte

**Affiliations:** ^1^Department of Medical Surgical Sciences and Biotechnologies, Sapienza University of Rome, Rome, Italy; ^2^Mediterranea Cardiocentro, Naples, Italy; ^3^National Heart and Lung Institute, Imperial College London, London, United Kingdom; ^4^Australian Regenerative Medicine Institute, Monash University, Clayton, VIC, Australia; ^5^The Jackson Laboratory, Bar Harbor, ME, United States

**Keywords:** fibrosis, inflammation, tissue damage, tissue repair, fibroblasts, stromal cells, leucocytes

Mammalian tissues react to damage by activating the highly specialized response of wound healing. Acute inflammation is the first and fastest mechanism activated in the damaged area, as a fundamental protective and transitional response with the purpose of progressive activation of endogenous tissue cells for reparative activities (Reinke and Sorg, 2012; Sattler, 2017). The balance between degree of damage, potency of the subsequent inflammatory wave, and the regenerative ability of resident cells determines the extent by which tissues can accomplish regeneration (for low-grade damage or in highly regenerative tissues, such as the liver) or fibrotic repair (for extensive damage or in poorly regenerative tissues, such as the myocardium). Persistent damage, complications, or repeated insults, can sustain the continuous or aberrant activation of repair pathways, leading to chronic inflammation and progressive tissue fibrosis (Sattler et al., 2017; Henderson et al., 2020). Despite the evolutionary advantage conferred to mammals by scarring as a rapid repair mechanism, biological and mechanical features of the original tissue architecture are disrupted in the scar area (Forbes and Rosenthal, 2014). Over time, chronic fibrosis thus leads to tissue adverse remodeling and impaired function. Persistent inflammation and fibrosis play a major role in a wide range of diseases, accounting for an increasingly large fraction of morbidity and mortality worldwide (Henderson et al., 2020).

While recent advances have unveiled many environmental and genetic causes of fibrotic disorders (Spagnolo et al., 2014; Cutting, 2015), a better understanding of both ubiquitous and tissue-specific regulatory pathways, and cellular dynamics could help to design new targeted therapies, and even to identify the etiology of idiopathic disease.

Within this Research Topic, we have gathered several contributions on the cellular and molecular mediators of fibrosis, and the intertwined pathophysiological role of fibrosis and inflammation in different tissues and diseases, with particular emphasis on renal and cardiac fibrosis.

## Cellular and Molecular Mediators of Fibrosis

Fibroblasts are increasingly regarded as a complex cell type. In fact, it has become clear that multiple phenotypes and functional states can be identified in the stroma of organs, with significantly different functions (Forte et al., 2018, 2020; Farbehi et al., 2019; Muhl et al., 2020; Mohenska et al., 2021; Plikus et al., 2021). Here, Huang et al. contributed a critical review on the association between specific fibroblast genes with biological and functional definitions, as this link seems to be subtle and blurred in many different tissues. The complex topic of fibroblast phenotypes is further complicated under pro-fibrotic conditions by the trans-differentiation of endothelial cells into myofibroblasts by endothelial-to-mesenchymal transition—End-MT (Forte et al., 2017; Hulshoff et al., 2019). Giordo et al. contributed a review on the interesting relationship between non-coding RNAs, oxidative stress, and End-MT in fibrosis complications affecting the retina, kidneys, heart, and vessels during diabetes progression.

However, fibroblasts are not the only stromal cell type of interest for tissue fibrosis and repair (Forte et al., 2018; Farbehi et al., 2019). Tissue resident mesenchymal cells are described in multiple organs, where they are involved in homeostasis and disease (Lemos and Duffield, 2018). Theret et al. reviewed the literature about fibro-adipogenic progenitors in muscle integrity, extracellular matrix (ECM) deposition, and in response to injury, also analyzing their contribution to the pool of activated fibroblasts in muscle fibrosis and degenerative conditions. On a different perspective, cardiac cell therapy with mesenchymal cells holds promise for the treatment of myocardial infarction (Peruzzi et al., 2015; Pagano et al., 2019; Guo et al., 2020). Tang et al. described that thymosin-β4 overexpression in bone-marrow mesenchymal cells significantly increases their therapeutic potential on cardiac function in a rat model. This effect is mediated, among others, by a reduction of the scar area and decrease in collagen deposition in the infarcted myocardium, and by increased activation of the Hypoxia-Inducible Factor 1-alpha (HIF-1α) pathway.

One hallmark of fibrosis in multiple organs is excessive ECM deposition (Herrera et al., 2018). Leong et al. reviewed the literature about the key enzymes of ECM remodeling, matrix metalloproteinases (MMPs) and their tissue inhibitors, in systemic sclerosis, on the quest for novel therapeutic targets to treat tissue stiffening and loss of function. MMPs also regulate the bioavailability of humoral factors. Adu-Amankwaah et al. contributed a review on the multiple roles of A Disintegrin And Metalloproteinase 17 (ADAM17), which is emerging as a core regulatory factor in inflammation and fibrosis. In fact, ADAM17 can activate multiple cytokines and pro-fibrotic factors (e.g., Tumor Necrosis Factor alpha—TNFα, soluble InterLeukin 6 Receptor—sIL-6R, Amphiregulin—AREG) in cardiac fibrosis and in catecholamine stress during heart failure progression, highlighting potential therapeutic targets. Moreover, cell–ECM interaction and crosstalk are responsible for the effects of tissue stiffening in fibrotic diseases on both parenchymal and stromal cells. Key transducers of ECM stiffening are integrins, and Xu et al. studied the integrin family expression in skin tissue of systemic sclerosis patients from the Gene Expression Omnibus-GEO database, describing the upregulation of several integrins involved in ECM turnover, leucocyte extravasation, Transforming Growth Factor beta 1 (TGF-β1) signaling, and immune cell activation. These results highlight the strong relationship between ECM, resident cells, and immune cells in tissue remodeling and fibrosis.

Fuentes-Calvo et al. provided another contribution to the understanding of molecular mechanisms of fibroblast activation, by describing the role of RAt Sarcoma virus protein (RAS) activator Son Of Sevenless 1 (SOS1) in murine embryonic fibroblasts. Their results show that SOS1 mediates the response to TGF-β1 through AK strain Transforming (AKT) and Extracellular signal-Regulated Kinase (ERK), particularly by mediating proliferation and migration of activated fibroblasts.

Pandita et al. contributed a study on the relationship between mitochondrial voltage, as an indicator of oxidative phosphorylation levels, and the progression of fibrosis in the liver. They showed mechanistic association between the upregulation of mitochondrial electron transport chain enzymes, oxygen consumption, and the activation of the AMP-activated protein Kinase (AMPK) pathway, providing an interesting link between metabolism, hepatic insult responses, and early signs of fibrosis in the liver.

## Cardiac Fibrosis

Myocardial fibrosis is involved in multiple cardiac diseases, and is fueled by continuous cardiomyocyte death, inflammatory cell recruitment, and direct activation of resident fibroblasts (Schirone et al., 2017; Forte et al., 2018, 2020; Panahi et al., 2018). Multiple signaling pathways are known to control fibroblast behavior (Plikus et al., 2021). Xie et al. investigated the role of High-Mobility Group A1 protein (HMGA1) in cardiac fibroblast activation, showing how it is overexpressed in an isoproterenol-treated mouse model, and that it mediates fibrosis via FOrkhead boX protein O1 (FOXO1). Conversely, its knockdown inhibits fibroblast responses to TGF-β1, thus identifying a novel molecular target for possible anti-fibrotic treatments in the heart.

Platelet-derived growth factors (PDGFs) are important mediators of inflammation, angiogenesis, and fibroblasts activation, and represent possible therapeutic targets against myocardial remodeling. Kalra et al. reviewed the literature concerning PDGF signaling mechanisms and beneficial effects in cardiac injury, highlighting the potentials and pitfalls for translational research. Concerning the identification of specific markers for myocardial injury, Cimini et al. contributed a review on podoplanin, a novel marker upregulated in multiple cell types after infarction. During homeostasis, podoplanin is expressed by lymphatic endothelial cells, but after injury many cell types upregulate its expression, including fibroblasts and hematopoietic cells. The authors provide an overview of several studies evidencing a wide range of possible interactions mediated by podoplanin among different cell compartments.

Concerning novel synergistic therapeutic approaches for the heart, Hou et al. studied how estradiol supplementation in rats during isoproterenol-induced stress can prevent cardiac dysfunction and maladaptive myocardial hypertrophy. In particular, these effects were shown to be mediated by β2-adrenoceptors and the resolution of inflammation due to an increased anti-inflammatory macrophage phenotype, evidencing an interesting endocrine/immune-modulatory circuit in the heart. Adzika et al. studied a possible combined therapy with forskolin (i.e., an adenylyl cyclase activator able to modulate cardiac and inflammatory responses) and amlexanox [i.e., a G protein-coupled Receptor Kinase 5 (GRK5) inhibitor of adaptive immune responses] to prevent pathological cardiac hypertrophy in mice. This treatment was effective at preventing left ventricular systolic dysfunction during isoproterenol-induced chronic stress by attenuating maladaptive myocardial hypertrophy, fibrosis, and inflammatory responses. Finally, Yang et al. studied how Receptor Interacting Protein kinase 2 (RIP2) is upregulated in failing hearts, both in human patient samples and murine models of aortic banding, as well as in angiotensin II-treated neonatal rat cardiomyocytes. RIP2 overexpression indeed aggravates cardiac remodeling through phosphorylation of targets such as TGFβ-Activated Kinase 1 (TAK1), p38 mitogen-activated protein kinase (p38MAPK), and Jun N-terminal Kinases (JNK1/2). Thus, inhibiting RIP2 or its targets may inhibit cardiac remodeling, representing another potential preventive strategy in cardiology.

## Renal Fibrosis

Fibrosis and inflammation are also significantly involved in kidney pathophysiology and progressive functional impairment, particularly in chronic kidney disease (Panizo et al., 2021). Cao et al. reviewed the literature on the role of lymphocytes in kidney fibrosis and disease, discussing how different subsets play different and antagonistic roles: in fact, T-helper cells and innate-like lymphocytes exert mainly pathogenic roles, while CD4^−^/CD8^−^ double negative and T-reg cells are considered protective. Further elucidation of the different roles of lymphocytes in renal injury and fibrosis may guide the discovery of novel specific therapeutics. Instead, Fang et al. focused their attention on diabetic kidney disease and the gut microbiota: they show that the microbiota metabolite trimethylamine N-oxide accelerates kidney dysfunction by activating the inflammasome and releasing pro-inflammatory cytokines in a high-fat diet/low-dose streptozotocin-induced rat model of diabetes, providing new perspectives on the pathogenesis and potential treatment of diabetic kidney disease.

Zhang et al. reported a novel protective mechanism of the traditional Chinese medicine compound called Qishen Yiqi Dripping Pill on diabetic nephropathy. In a high-fat diet/streptozotocin rat model of diabetes this treatment yielded improved kidney function and reduced fibrosis; moreover, they evidenced inhibition of the Wingless-iNTegration—Wnt/β-catenin and the TGF-β/Smad2 signaling pathways, thus highlighting a potential direct effect on activation of tissue fibrosis. Regarding other related mechanisms of injury, Jia et al. described that attenuation of DNA damage and senescence in tubular cells by the NAD+ precursor nicotinamide mononucleotide (NMN) may exert a protective effect against acute kidney injury; they confirmed this therapeutic and preventive potential both *in vitro* and *in vivo*, in an ischemia-reperfusion injury mouse model showing cell protection and reduced inflammation.

Concerning direct interference with mechanisms of fibroblast activation and fibrosis, Xie et al. showed that exogenous transfection of the Bone Morphogenetic Protein binding Endothelial Regulator (BMPER) in a mouse model of renal fibrosis blocks fundamental mechanisms of fibroblast activation mediated by TGF-β1, such as epithelial-to-mesenchymal transition and tubular cells trans-differentiation, proposing another novel therapeutic strategy against renal fibrosis. In this regard, Wu et al. identified the circular RNA circHIPK3 as a possible novel therapeutic target for renal fibrosis. They found that circHIPK3 is upregulated in a mouse model of induced renal fibrosis. The study demonstrated that circHIPK3 reduces the activity of miRNA-30a, a known negative regulator of profibrotic genes, including TGF-β1, fibronectin, and collagen 1. CircHIPK3 sequesters miRNA-30a in the cytoplasm of tubular epithelial cells by directly binding to it, and this promotes the expression of profibrotic genes and the progression of renal fibrosis.

Inflammation and fibrosis are closely connected to alterations in the composition and properties of the ECM (Pesce et al., 2017; Herrera et al., 2018). As for the heart, the mechanisms by which ECM stiffening may aggravate renal fibrosis are still under investigation. Fu et al. reported that enhanced substrate stiffness can activate mesangial cells toward fibrotic features through the Piezo1, p38MAPK, and Yes-Associated Protein (YAP) pathway. They showed both *in vitro* and *in vivo* in a unilateral ureteral occlusion model in mice, that Piezo1 knockdown can counteract this activation, as well as fibrosis progression, suggesting a novel mechanosensing-based strategy against renal fibrosis. Another contribution on mechanosensing is focused on diabetes and the vasculature. Diabetes represents the starting condition triggering multiple organ damage (Frati et al., 2017; Cosentino et al., 2020). Ortillon et al. reported that YAP signaling is activated *in vitro* in endothelial cells by exposure to high glucose, and *in vivo* in the vessels of db/db mice. Its activation is potentiated under flow stress, evidencing a novel role in enhancing diabetic inflammatory signals in the vasculature.

## Fibrosis Modeling and Drug Testing

Research into novel treatments targeting fibrosis greatly benefits from advancements in relevant and reproducible animal models, as well as *in vitro* systems with increasing physiological relevance (Lagares and Hinz, 2021). As part of this article collection, Palano et al. summarized and discussed available assays to study cardiac fibrosis, with emphasis on the most recent innovations in multi-cellular 3D cultures and computational methods for scaling up and boosting preclinical and translational research. Bao et al., on the other hand, focused their review on the analysis and comparison of different animal models of liver fibrosis and their ability to mimic different etiologies and pathogenic mechanisms. They also presented the most recent advances in liver organoid cultures as relevant alternatives to *in vivo* testing.

Looking for novel anti-inflammatory and immunosuppressive therapeutic options, Pryimak et al. reviewed the growing body of literature suggesting that cannabinoids and *Cannabis sativa* extracts may exert anti-fibrotic effects at multiple levels and in multiple organs. In this review, the authors suggest the introduction of cannabinoids for the prevention and treatment of fibrosis as a versatile and widely applicable therapeutic strategy.

In conclusion, the present Research Topic has collected a significant amount of novel and interconnected contributions on the multifaceted roles of inflammation and fibrosis in different tissues and diseases. The increasing knowledge in the field is paving the way towards multi-target therapeutic strategies against chronic diseases, as well as to novel treatments adaptable to various organs sharing similar pathogenetic pathways.

## Author Contributions

All authors listed have made a substantial, direct, and intellectual contribution to the work and approved it for publication.

## Funding

IC was supported by grant # RG11916B85CDBF76 from Sapienza University of Rome. GM-N was supported by a Heart Foundation Future Leader Fellowship (102036) and Australian Research Council Discovery Project Grants (DP190101475, DP210102134). The Australian Regenerative Medicine Institute is supported by grants from the State Government of Victoria and the Australian Government. SS was generously supported by the British Heart Foundation (PG/16/93/32345).

## Conflict of Interest

The authors declare that the research was conducted in the absence of any commercial or financial relationships that could be construed as a potential conflict of interest.

## Publisher's Note

All claims expressed in this article are solely those of the authors and do not necessarily represent those of their affiliated organizations, or those of the publisher, the editors and the reviewers. Any product that may be evaluated in this article, or claim that may be made by its manufacturer, is not guaranteed or endorsed by the publisher.
